# {*meso*-Tetra­kis[*p*-(hept­yloxy)phen­yl]­porphyrinato}silver(II)

**DOI:** 10.1107/S160053681103385X

**Published:** 2011-08-27

**Authors:** Jun-Xu Liao, Hong-Bin Zhao, De-Liang Yang, Liang Chen, Bang-Ying Wang

**Affiliations:** a Environmental Engineering, Dongguan University of Technology, Guangdong, 523808, People’s Republic of China; b Cleaner Production Center, Dongguan University of Technology, Guangdong, 523808, People’s Republic of China; c Department of Organic Chemistry, the College of Chemistry, Xiangtan University, Hunan, 411105, People’s Republic of China

## Abstract

The title compound, [Ag(C_72_H_84_N_4_O_4_)], crystallizes with the Ag^II^ cation on a centre of symmetry. The macrocyclic 24-membered ring core is planar with a mean deviation of 0.0311 (15) Å and the four-coordinate Ag^II^ cation fits into its center, at 2.0814 (19) and 2.0872 (19) Å, from the surrounding pyrrole-N atoms, in agreement with what is found in related compounds. The *p*-heptyl­oxyphenyl groups are rotated 75.51 (5) and 84.45 (8)° with respect to the porphyrin mean plane, due to steric hindrance with the pyrrole-H atoms of the macrocycle.

## Related literature

For background information on metalloporphyrins and their derivatives, see: Fu *et al.* (2009 ▶); Jurow *et al.* (2010 ▶); Taniguchi & Lindsey (2010 ▶); Zenkevich *et al.* (2001 ▶). For related structures, see: Scheidt *et al.* (1986 ▶); Xu *et al.* (2007 ▶).
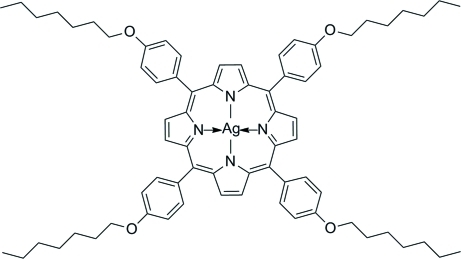


## Experimental

### 

#### Crystal data

[Ag(C_72_H_84_N_4_O_4_)]
*M* *_r_* = 1177.30
Monoclinic, 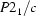

*a* = 15.850 (1) Å
*b* = 19.1896 (12) Å
*c* = 10.3285 (7) Å
β = 91.724 (1)°
*V* = 3140.0 (4) Å^3^
*Z* = 2
Mo *K*α radiation
μ = 0.37 mm^−1^
*T* = 185 K
0.24 × 0.17 × 0.10 mm

#### Data collection

Bruker APEX CCD diffractometer
Absorption correction: multi-scan (*SADABS*; Sheldrick, 2004 ▶) *T*_min_ = 0.916, *T*_max_ = 0.964
18310 measured reflections
5544 independent reflections
4385 reflections with *I* > 2σ(*I*)
*R*_int_ = 0.035

#### Refinement

*R*[*F* ^2^ > 2σ(*F* ^2^)] = 0.036
*wR*(*F* ^2^) = 0.091
*S* = 1.02
5544 reflections
369 parameters
H-atom parameters constrained
Δρ_max_ = 0.53 e Å^−3^
Δρ_min_ = −0.20 e Å^−3^

### 

Data collection: *SMART* (Bruker, 2002 ▶); cell refinement: *SAINT* (Bruker, 2002 ▶); data reduction: *SAINT*; program(s) used to solve structure: *SHELXS97* (Sheldrick, 2008 ▶); program(s) used to refine structure: *SHELXL97* (Sheldrick, 2008 ▶) and *PLATON* (Spek, 2009 ▶); molecular graphics: *SHELXTL* (Sheldrick, 2008 ▶); software used to prepare material for publication: *SHELXTL*.

## Supplementary Material

Crystal structure: contains datablock(s) global, I. DOI: 10.1107/S160053681103385X/bg2416sup1.cif

Structure factors: contains datablock(s) I. DOI: 10.1107/S160053681103385X/bg2416Isup2.hkl

Additional supplementary materials:  crystallographic information; 3D view; checkCIF report

## Figures and Tables

**Fig. 1.**
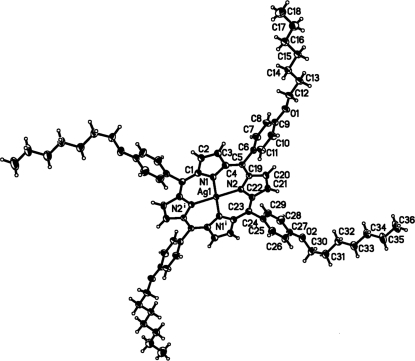
A view of (I), with the atom-labeling scheme and 50% probability displacement ellipsoids. Symmetry codes: (i) -x, -y, -z+2.

## supplementary crystallographic information

### Comment

Porphyrins, metalloporphyrins, and their derivatives are applied in many fields, such as biomimetic catalysts (Fu *et al.*, 2009), molecular electronic components (Jurow *et al.*, 2010), artificial photosynthesis (Taniguchi *et al.*, 2010) or electron transfer and energy migration (Zenkevich *et al.*, 2001). In this paper, the structure of Silver(II)*meso*-tetrakis[*p*-(heptyloxy)phenyl]porphyrinate (I) is reported.

The compound crystallizes with the Ag^II^ cation in a centre of symmetry (Fig. 1). The macrocyclic 24-membered ring core is planar with a mean deviation of 0.0311 (15) Å and the four coordinate Ag^II^ ion fits into its center, at 2.0814 (19) and 2.0872 (19) Å, from the surrouding pyrrole N atoms, in agreement with what found in related compounds (Scheidt *et al.*, 1986; Xu *et al.*, 2007).

The *p*-heptyloxyphenyl groups are rotated at angles of 75.51 (5)° and 84.45 (8)° with respect to the porphyrin mean plane, due to steric hindrance with the pyrrole-H atoms of the macrocycle.

### Experimental

0.03mmol meso-tetrakis[p-(heptyloxy)phenyl] porphyrin and 0.06mmol AgNO_3_ were dissolved in 20 ml chloroform, refluxed for 6 hours, and the solvent was removed by a rotary evaporator, the residue was purified by column chromatography with chloroform, then recrystallized from a methanol/chloroform solution, and a purple solid was obtained (yield=23%). Single crystals were obtained from recrystallization from a dichloromethane solution at room temperature.

### Refinement

H atoms were placed in calculated positions (C—H = 0.95, 0.98 or 0.99 Å) and refined in riding mode, with *U*_iso_(H) = *xU*_eq_(C), where *x* = 1.5 for methyl and 1.2 for all other H atoms.

A Platon run (Spek, 2009) detects solvent accessible voids of 78 Å^3^, which indicate that the structure may contain disordered solvent molecules (dichloromethane). However, efforts to locate the solvent molecules failed because the residual electron density is small (the highest peak of residual density is 0.529 e Å^-3^).

### Crystal data

**Table:**

[Ag(C_72_H_84_N_4_O_4_)]	*F*(000) = 1246
*M**_r_* = 1177.30	*D*_x_ = 1.245 Mg m^−^^3^
Monoclinic, *P*2_1_/*c*	Mo *K*α radiation, λ = 0.71073 Å
Hall symbol: -P 2ybc	Cell parameters from 5112 reflections
*a* = 15.850 (1) Å	θ = 2.2–24.8°
*b* = 19.1896 (12) Å	µ = 0.37 mm^−^^1^
*c* = 10.3285 (7) Å	*T* = 185 K
β = 91.724 (1)°	Block, purple
*V* = 3140.0 (4) Å^3^	0.24 × 0.17 × 0.10 mm
*Z* = 2	

### Data collection

**Table:**

Bruker APEX CCD diffractometer	5544 independent reflections
Radiation source: fine-focus sealed tube	4385 reflections with *I* > 2σ(*I*)
graphite	*R*_int_ = 0.035
φ and ω scans	θ_max_ = 25.0°, θ_min_ = 1.7°
Absorption correction: multi-scan (*SADABS*; Sheldrick, 2004)	*h* = −17→18
*T*_min_ = 0.916, *T*_max_ = 0.964	*k* = −22→19
18310 measured reflections	*l* = −12→12

### Refinement

**Table:**

Refinement on *F*^2^	Primary atom site location: structure-invariant direct methods
Least-squares matrix: full	Secondary atom site location: difference Fourier map
*R*[*F*^2^ > 2σ(*F*^2^)] = 0.036	Hydrogen site location: inferred from neighbouring sites
*wR*(*F*^2^) = 0.091	H-atom parameters constrained
*S* = 1.02	*w* = 1/[σ^2^(*F*_o_^2^) + (0.0483*P*)^2^ + 0.7718*P*] where *P* = (*F*_o_^2^ + 2*F*_c_^2^)/3
5544 reflections	(Δ/σ)_max_ < 0.001
369 parameters	Δρ_max_ = 0.53 e Å^−^^3^
0 restraints	Δρ_min_ = −0.20 e Å^−^^3^

### Special details

**Table:**

Geometry. All e.s.d.'s (except the e.s.d. in the dihedral angle between two l.s. planes) are estimated using the full covariance matrix. The cell e.s.d.'s are taken into account individually in the estimation of e.s.d.'s in distances, angles and torsion angles; correlations between e.s.d.'s in cell parameters are only used when they are defined by crystal symmetry. An approximate (isotropic) treatment of cell e.s.d.'s is used for estimating e.s.d.'s involving l.s. planes.
Refinement. Refinement of *F*^2^ against ALL reflections. The weighted *R*-factor *wR* and goodness of fit *S* are based on *F*^2^, conventional *R*-factors *R* are based on *F*, with *F* set to zero for negative *F*^2^. The threshold expression of *F*^2^ > σ(*F*^2^) is used only for calculating *R*-factors(gt) *etc*. and is not relevant to the choice of reflections for refinement. *R*-factors based on *F*^2^ are statistically about twice as large as those based on *F*, and *R*- factors based on ALL data will be even larger.

### Fractional atomic coordinates and isotropic or equivalent isotropic displacement parameters (Å^2^)

**Table:**

	*x*	*y*	*z*	*U*_iso_*/*U*_eq_	
Ag1	0.0000	0.0000	1.0000	0.02274 (9)	
C1	−0.17114 (15)	0.03711 (13)	1.1183 (2)	0.0268 (6)	
C2	−0.22273 (16)	0.09570 (13)	1.1505 (2)	0.0307 (6)	
H2	−0.2766	0.0938	1.1884	0.037*	
C3	−0.18104 (16)	0.15392 (14)	1.1173 (2)	0.0302 (6)	
H3	−0.2003	0.2004	1.1272	0.036*	
C4	−0.10181 (15)	0.13291 (13)	1.0637 (2)	0.0267 (6)	
C5	−0.03796 (16)	0.17749 (12)	1.0229 (2)	0.0242 (5)	
C6	−0.05259 (15)	0.25455 (12)	1.0355 (2)	0.0254 (5)	
C7	−0.10737 (17)	0.29051 (14)	0.9522 (2)	0.0347 (6)	
H7	−0.1365	0.2661	0.8845	0.042*	
C8	−0.12023 (17)	0.36174 (14)	0.9664 (2)	0.0353 (6)	
H8	−0.1582	0.3854	0.9087	0.042*	
C9	−0.07828 (16)	0.39834 (13)	1.0637 (2)	0.0284 (6)	
C10	−0.02294 (18)	0.36346 (14)	1.1466 (2)	0.0361 (7)	
H10	0.0069	0.3880	1.2134	0.043*	
C11	−0.01107 (18)	0.29203 (13)	1.1314 (2)	0.0354 (6)	
H11	0.0269	0.2684	1.1892	0.042*	
C12	−0.04960 (18)	0.50778 (12)	1.1669 (3)	0.0341 (6)	
H12A	0.0109	0.5097	1.1459	0.041*	
H12B	−0.0545	0.4855	1.2528	0.041*	
C13	−0.08601 (17)	0.58049 (13)	1.1696 (3)	0.0339 (6)	
H13A	−0.0896	0.5991	1.0802	0.041*	
H13B	−0.0476	0.6110	1.2214	0.041*	
C14	−0.17338 (17)	0.58243 (13)	1.2270 (3)	0.0337 (6)	
H14A	−0.2116	0.5524	1.1739	0.040*	
H14B	−0.1696	0.5623	1.3153	0.040*	
C15	−0.21219 (17)	0.65466 (14)	1.2351 (3)	0.0350 (6)	
H15A	−0.2249	0.6720	1.1463	0.042*	
H15B	−0.1707	0.6868	1.2765	0.042*	
C16	−0.29271 (18)	0.65544 (14)	1.3115 (3)	0.0383 (7)	
H16A	−0.3327	0.6213	1.2724	0.046*	
H16B	−0.2790	0.6399	1.4011	0.046*	
C17	−0.3360 (2)	0.72544 (17)	1.3171 (3)	0.0521 (8)	
H17A	−0.3525	0.7400	1.2280	0.062*	
H17B	−0.2954	0.7603	1.3527	0.062*	
C18	−0.4137 (2)	0.72520 (19)	1.3994 (4)	0.0679 (10)	
H18A	−0.4538	0.6902	1.3659	0.102*	
H18B	−0.4403	0.7713	1.3961	0.102*	
H18C	−0.3973	0.7139	1.4892	0.102*	
C19	0.04070 (16)	0.15704 (12)	0.9737 (2)	0.0240 (5)	
C20	0.10418 (16)	0.20316 (13)	0.9264 (2)	0.0291 (6)	
H20	0.1026	0.2526	0.9284	0.035*	
C21	0.16623 (16)	0.16374 (13)	0.8789 (2)	0.0281 (6)	
H21	0.2160	0.1804	0.8402	0.034*	
C22	0.14361 (15)	0.09178 (13)	0.8972 (2)	0.0253 (6)	
C23	0.19112 (15)	0.03383 (14)	0.8601 (2)	0.0264 (6)	
C24	0.26926 (16)	0.04802 (13)	0.7844 (2)	0.0284 (6)	
C25	0.26415 (17)	0.04737 (15)	0.6514 (3)	0.0379 (7)	
H25	0.2115	0.0372	0.6092	0.045*	
C26	0.33407 (17)	0.06126 (15)	0.5767 (3)	0.0397 (7)	
H26	0.3292	0.0597	0.4849	0.048*	
C27	0.41000 (17)	0.07728 (13)	0.6369 (3)	0.0326 (6)	
C28	0.41643 (17)	0.07760 (16)	0.7708 (3)	0.0428 (7)	
H28	0.4690	0.0880	0.8130	0.051*	
C29	0.34655 (17)	0.06290 (15)	0.8436 (3)	0.0388 (7)	
H29	0.3519	0.0630	0.9354	0.047*	
C30	0.47894 (19)	0.09010 (16)	0.4349 (3)	0.0432 (7)	
H30A	0.4315	0.1190	0.4008	0.052*	
H30B	0.4693	0.0414	0.4062	0.052*	
C31	0.56106 (19)	0.11666 (15)	0.3838 (3)	0.0466 (8)	
H31A	0.5638	0.1047	0.2907	0.056*	
H31B	0.6084	0.0927	0.4298	0.056*	
C32	0.57217 (18)	0.19524 (15)	0.3996 (3)	0.0431 (7)	
H32A	0.5192	0.2186	0.3707	0.052*	
H32B	0.5817	0.2059	0.4927	0.052*	
C33	0.64472 (18)	0.22525 (15)	0.3246 (3)	0.0437 (7)	
H33A	0.6986	0.2064	0.3608	0.052*	
H33B	0.6390	0.2100	0.2331	0.052*	
C34	0.6478 (2)	0.30437 (16)	0.3290 (3)	0.0500 (8)	
H34A	0.5923	0.3228	0.2990	0.060*	
H34B	0.6573	0.3192	0.4201	0.060*	
C35	0.7154 (2)	0.33636 (16)	0.2479 (4)	0.0569 (9)	
H35A	0.7076	0.3200	0.1575	0.068*	
H35B	0.7713	0.3198	0.2805	0.068*	
C36	0.7149 (3)	0.41504 (18)	0.2490 (4)	0.0837 (13)	
H36A	0.6608	0.4319	0.2128	0.126*	
H36B	0.7609	0.4325	0.1967	0.126*	
H36C	0.7225	0.4317	0.3383	0.126*	
N1	−0.09901 (12)	0.06134 (10)	1.06458 (18)	0.0258 (5)	
N2	0.06710 (12)	0.08969 (10)	0.95571 (18)	0.0236 (4)	
O1	−0.09551 (11)	0.46820 (9)	1.07045 (16)	0.0327 (4)	
O2	0.48278 (11)	0.09324 (10)	0.57368 (18)	0.0400 (5)	

### Atomic displacement parameters (Å^2^)

**Table:**

	*U*^11^	*U*^22^	*U*^33^	*U*^12^	*U*^13^	*U*^23^
Ag1	0.02106 (15)	0.01858 (15)	0.02898 (14)	0.00032 (12)	0.00751 (10)	0.00036 (11)
C1	0.0219 (14)	0.0264 (15)	0.0324 (13)	0.0016 (11)	0.0069 (11)	−0.0005 (11)
C2	0.0256 (14)	0.0273 (15)	0.0399 (14)	0.0000 (12)	0.0114 (11)	−0.0009 (11)
C3	0.0270 (14)	0.0256 (14)	0.0386 (14)	0.0045 (12)	0.0089 (11)	−0.0017 (11)
C4	0.0245 (14)	0.0254 (15)	0.0303 (13)	0.0028 (11)	0.0025 (10)	−0.0006 (10)
C5	0.0265 (14)	0.0195 (13)	0.0267 (12)	0.0012 (11)	0.0036 (10)	−0.0009 (10)
C6	0.0243 (13)	0.0210 (13)	0.0313 (13)	0.0003 (11)	0.0064 (10)	0.0012 (10)
C7	0.0372 (16)	0.0267 (15)	0.0399 (14)	0.0015 (12)	−0.0061 (12)	−0.0058 (12)
C8	0.0391 (16)	0.0251 (15)	0.0411 (15)	0.0078 (13)	−0.0090 (12)	0.0004 (12)
C9	0.0327 (15)	0.0202 (13)	0.0326 (13)	0.0030 (11)	0.0063 (11)	0.0010 (11)
C10	0.0457 (17)	0.0268 (15)	0.0355 (14)	0.0032 (13)	−0.0059 (12)	−0.0079 (11)
C11	0.0447 (17)	0.0249 (15)	0.0361 (14)	0.0097 (13)	−0.0060 (12)	−0.0010 (11)
C12	0.0394 (16)	0.0235 (15)	0.0394 (14)	0.0003 (12)	0.0022 (12)	−0.0067 (11)
C13	0.0379 (16)	0.0238 (15)	0.0404 (15)	0.0003 (12)	0.0055 (12)	−0.0036 (11)
C14	0.0385 (16)	0.0241 (15)	0.0387 (14)	−0.0030 (12)	0.0059 (12)	−0.0013 (11)
C15	0.0376 (17)	0.0255 (15)	0.0423 (15)	−0.0006 (13)	0.0102 (12)	−0.0029 (12)
C16	0.0392 (17)	0.0338 (16)	0.0422 (15)	−0.0006 (13)	0.0069 (13)	−0.0012 (13)
C17	0.046 (2)	0.0436 (19)	0.067 (2)	0.0084 (16)	0.0201 (16)	−0.0013 (16)
C18	0.067 (3)	0.059 (2)	0.080 (2)	0.015 (2)	0.036 (2)	0.0017 (19)
C19	0.0279 (14)	0.0190 (13)	0.0254 (12)	−0.0008 (11)	0.0030 (10)	−0.0001 (10)
C20	0.0293 (15)	0.0223 (14)	0.0360 (13)	−0.0010 (12)	0.0052 (11)	0.0014 (11)
C21	0.0238 (14)	0.0250 (14)	0.0359 (14)	−0.0045 (11)	0.0075 (11)	0.0039 (11)
C22	0.0239 (14)	0.0229 (14)	0.0294 (12)	−0.0014 (11)	0.0038 (10)	0.0006 (10)
C23	0.0212 (14)	0.0285 (15)	0.0296 (13)	−0.0007 (12)	0.0049 (10)	−0.0015 (11)
C24	0.0266 (14)	0.0205 (14)	0.0387 (14)	−0.0001 (11)	0.0092 (11)	−0.0015 (11)
C25	0.0267 (15)	0.0478 (18)	0.0393 (15)	−0.0075 (13)	0.0039 (12)	0.0008 (13)
C26	0.0344 (16)	0.0507 (19)	0.0345 (14)	−0.0066 (14)	0.0078 (12)	0.0040 (13)
C27	0.0293 (15)	0.0251 (15)	0.0441 (15)	−0.0008 (12)	0.0146 (12)	−0.0003 (12)
C28	0.0251 (15)	0.055 (2)	0.0480 (16)	−0.0101 (14)	0.0058 (12)	−0.0047 (14)
C29	0.0316 (16)	0.0503 (19)	0.0350 (14)	−0.0076 (14)	0.0072 (12)	−0.0063 (13)
C30	0.0437 (18)	0.0407 (18)	0.0463 (16)	−0.0043 (15)	0.0211 (14)	0.0019 (14)
C31	0.0451 (19)	0.0381 (18)	0.0581 (18)	0.0015 (15)	0.0265 (15)	0.0034 (15)
C32	0.0376 (17)	0.0376 (17)	0.0551 (17)	0.0015 (14)	0.0189 (14)	0.0007 (14)
C33	0.0329 (16)	0.0355 (17)	0.0637 (19)	0.0010 (14)	0.0175 (14)	0.0060 (14)
C34	0.047 (2)	0.0381 (18)	0.066 (2)	−0.0011 (15)	0.0148 (16)	0.0048 (15)
C35	0.046 (2)	0.0369 (19)	0.088 (2)	−0.0063 (16)	0.0157 (18)	0.0155 (17)
C36	0.083 (3)	0.040 (2)	0.130 (4)	−0.014 (2)	0.035 (3)	0.011 (2)
N1	0.0238 (11)	0.0196 (11)	0.0346 (11)	0.0014 (9)	0.0092 (9)	0.0005 (9)
N2	0.0214 (11)	0.0199 (11)	0.0300 (10)	0.0016 (9)	0.0071 (8)	0.0000 (8)
O1	0.0418 (12)	0.0206 (9)	0.0355 (10)	0.0054 (8)	−0.0016 (8)	−0.0037 (8)
O2	0.0299 (11)	0.0412 (12)	0.0499 (11)	−0.0050 (9)	0.0179 (9)	0.0035 (9)

### Geometric parameters (Å, °)

**Table:**

Ag1—N2	2.0814 (19)	C18—H18B	0.9800
Ag1—N2^i^	2.0815 (19)	C18—H18C	0.9800
Ag1—N1^i^	2.0871 (19)	C19—N2	1.373 (3)
Ag1—N1	2.0872 (19)	C19—C20	1.436 (3)
C1—N1	1.367 (3)	C20—C21	1.345 (3)
C1—C23^i^	1.417 (4)	C20—H20	0.9500
C1—C2	1.435 (3)	C21—C22	1.441 (3)
C2—C3	1.348 (3)	C21—H21	0.9500
C2—H2	0.9500	C22—N2	1.371 (3)
C3—C4	1.445 (3)	C22—C23	1.403 (3)
C3—H3	0.9500	C23—C1^i^	1.417 (4)
C4—N1	1.374 (3)	C23—C24	1.509 (3)
C4—C5	1.400 (3)	C24—C25	1.373 (3)
C5—C19	1.416 (3)	C24—C29	1.382 (4)
C5—C6	1.503 (3)	C25—C26	1.395 (3)
C6—C11	1.376 (3)	C25—H25	0.9500
C6—C7	1.387 (3)	C26—C27	1.372 (4)
C7—C8	1.390 (4)	C26—H26	0.9500
C7—H7	0.9500	C27—O2	1.377 (3)
C8—C9	1.380 (3)	C27—C28	1.384 (4)
C8—H8	0.9500	C28—C29	1.386 (4)
C9—O1	1.370 (3)	C28—H28	0.9500
C9—C10	1.380 (3)	C29—H29	0.9500
C10—C11	1.393 (4)	C30—O2	1.435 (3)
C10—H10	0.9500	C30—C31	1.508 (4)
C11—H11	0.9500	C30—H30A	0.9900
C12—O1	1.434 (3)	C30—H30B	0.9900
C12—C13	1.510 (3)	C31—C32	1.526 (4)
C12—H12A	0.9900	C31—H31A	0.9900
C12—H12B	0.9900	C31—H31B	0.9900
C13—C14	1.523 (4)	C32—C33	1.519 (4)
C13—H13A	0.9900	C32—H32A	0.9900
C13—H13B	0.9900	C32—H32B	0.9900
C14—C15	1.520 (3)	C33—C34	1.520 (4)
C14—H14A	0.9900	C33—H33A	0.9900
C14—H14B	0.9900	C33—H33B	0.9900
C15—C16	1.521 (4)	C34—C35	1.510 (4)
C15—H15A	0.9900	C34—H34A	0.9900
C15—H15B	0.9900	C34—H34B	0.9900
C16—C17	1.510 (4)	C35—C36	1.510 (4)
C16—H16A	0.9900	C35—H35A	0.9900
C16—H16B	0.9900	C35—H35B	0.9900
C17—C18	1.518 (4)	C36—H36A	0.9800
C17—H17A	0.9900	C36—H36B	0.9800
C17—H17B	0.9900	C36—H36C	0.9800
C18—H18A	0.9800		
			
N2—Ag1—N2^i^	180.0	N2—C19—C20	108.3 (2)
N2—Ag1—N1^i^	90.12 (7)	C5—C19—C20	125.8 (2)
N2^i^—Ag1—N1^i^	89.88 (7)	C21—C20—C19	107.8 (2)
N2—Ag1—N1	89.88 (7)	C21—C20—H20	126.1
N2^i^—Ag1—N1	90.12 (7)	C19—C20—H20	126.1
N1^i^—Ag1—N1	180.00 (7)	C20—C21—C22	107.6 (2)
N1—C1—C23^i^	125.8 (2)	C20—C21—H21	126.2
N1—C1—C2	108.5 (2)	C22—C21—H21	126.2
C23^i^—C1—C2	125.8 (2)	N2—C22—C23	125.9 (2)
C3—C2—C1	107.7 (2)	N2—C22—C21	108.2 (2)
C3—C2—H2	126.2	C23—C22—C21	125.9 (2)
C1—C2—H2	126.2	C22—C23—C1^i^	126.5 (2)
C2—C3—C4	107.7 (2)	C22—C23—C24	117.0 (2)
C2—C3—H3	126.1	C1^i^—C23—C24	116.5 (2)
C4—C3—H3	126.1	C25—C24—C29	118.0 (2)
N1—C4—C5	126.1 (2)	C25—C24—C23	119.5 (2)
N1—C4—C3	107.7 (2)	C29—C24—C23	122.6 (2)
C5—C4—C3	126.1 (2)	C24—C25—C26	121.8 (3)
C4—C5—C19	126.2 (2)	C24—C25—H25	119.1
C4—C5—C6	117.4 (2)	C26—C25—H25	119.1
C19—C5—C6	116.3 (2)	C27—C26—C25	119.5 (2)
C11—C6—C7	117.6 (2)	C27—C26—H26	120.2
C11—C6—C5	120.3 (2)	C25—C26—H26	120.2
C7—C6—C5	122.1 (2)	O2—C27—C26	124.8 (2)
C6—C7—C8	121.0 (2)	O2—C27—C28	115.8 (2)
C6—C7—H7	119.5	C26—C27—C28	119.4 (2)
C8—C7—H7	119.5	C27—C28—C29	120.3 (3)
C9—C8—C7	120.5 (2)	C27—C28—H28	119.8
C9—C8—H8	119.7	C29—C28—H28	119.8
C7—C8—H8	119.7	C28—C29—C24	120.9 (2)
O1—C9—C8	116.3 (2)	C28—C29—H29	119.5
O1—C9—C10	124.5 (2)	C24—C29—H29	119.5
C8—C9—C10	119.2 (2)	O2—C30—C31	108.9 (2)
C9—C10—C11	119.5 (2)	O2—C30—H30A	109.9
C9—C10—H10	120.2	C31—C30—H30A	109.9
C11—C10—H10	120.2	O2—C30—H30B	109.9
C6—C11—C10	122.1 (2)	C31—C30—H30B	109.9
C6—C11—H11	118.9	H30A—C30—H30B	108.3
C10—C11—H11	118.9	C30—C31—C32	113.2 (2)
O1—C12—C13	108.4 (2)	C30—C31—H31A	108.9
O1—C12—H12A	110.0	C32—C31—H31A	108.9
C13—C12—H12A	110.0	C30—C31—H31B	108.9
O1—C12—H12B	110.0	C32—C31—H31B	108.9
C13—C12—H12B	110.0	H31A—C31—H31B	107.7
H12A—C12—H12B	108.4	C33—C32—C31	114.0 (2)
C12—C13—C14	112.5 (2)	C33—C32—H32A	108.8
C12—C13—H13A	109.1	C31—C32—H32A	108.8
C14—C13—H13A	109.1	C33—C32—H32B	108.8
C12—C13—H13B	109.1	C31—C32—H32B	108.8
C14—C13—H13B	109.1	H32A—C32—H32B	107.7
H13A—C13—H13B	107.8	C34—C33—C32	112.8 (2)
C15—C14—C13	114.7 (2)	C34—C33—H33A	109.0
C15—C14—H14A	108.6	C32—C33—H33A	109.0
C13—C14—H14A	108.6	C34—C33—H33B	109.0
C15—C14—H14B	108.6	C32—C33—H33B	109.0
C13—C14—H14B	108.6	H33A—C33—H33B	107.8
H14A—C14—H14B	107.6	C35—C34—C33	114.3 (3)
C14—C15—C16	112.6 (2)	C35—C34—H34A	108.7
C14—C15—H15A	109.1	C33—C34—H34A	108.7
C16—C15—H15A	109.1	C35—C34—H34B	108.7
C14—C15—H15B	109.1	C33—C34—H34B	108.7
C16—C15—H15B	109.1	H34A—C34—H34B	107.6
H15A—C15—H15B	107.8	C34—C35—C36	113.5 (3)
C17—C16—C15	114.7 (2)	C34—C35—H35A	108.9
C17—C16—H16A	108.6	C36—C35—H35A	108.9
C15—C16—H16A	108.6	C34—C35—H35B	108.9
C17—C16—H16B	108.6	C36—C35—H35B	108.9
C15—C16—H16B	108.6	H35A—C35—H35B	107.7
H16A—C16—H16B	107.6	C35—C36—H36A	109.5
C16—C17—C18	113.3 (3)	C35—C36—H36B	109.5
C16—C17—H17A	108.9	H36A—C36—H36B	109.5
C18—C17—H17A	108.9	C35—C36—H36C	109.5
C16—C17—H17B	108.9	H36A—C36—H36C	109.5
C18—C17—H17B	108.9	H36B—C36—H36C	109.5
H17A—C17—H17B	107.7	C1—N1—C4	108.4 (2)
C17—C18—H18A	109.5	C1—N1—Ag1	125.72 (17)
C17—C18—H18B	109.5	C4—N1—Ag1	125.88 (16)
H18A—C18—H18B	109.5	C19—N2—C22	108.01 (19)
C17—C18—H18C	109.5	C19—N2—Ag1	126.08 (15)
H18A—C18—H18C	109.5	C22—N2—Ag1	125.83 (16)
H18B—C18—H18C	109.5	C9—O1—C12	117.12 (19)
N2—C19—C5	125.8 (2)	C27—O2—C30	117.0 (2)

Symmetry codes: (i) −*x*, −*y*, −*z*+2.
